# Marine-Derived Fungi as a Valuable Resource for Amylases Activity Screening

**DOI:** 10.3390/jof9070736

**Published:** 2023-07-09

**Authors:** Di Zhang, Lan Liu, Bi-Shuang Chen

**Affiliations:** 1 School of Marine Sciences, Sun Yat-Sen University, Zhuhai 519080, China; 2 Southern Marine Science and Engineering Guangdong Laboratory (Zhuhai), Zhuhai 519080, China

**Keywords:** marine-derived fungi, amylase, starch hydrolysis, enzyme activity, thermal and pH stability

## Abstract

Marine microbial enzymes including amylases are important in different industrial production due to their properties and applications. This study was focused on the screening of marine-derived fungi for amylase activities. First, we isolated a number of fungi from the sediments of the South China Sea. By the method of dish screening (in vitro), we subsequently obtained a series of amylase-producing fungal strains. The cell-lysate activities of amylases produced by marine fungi toward starch hydrolysis were achieved with the dinitrosalyicylic acid (DNS) method. In addition, the effect of pH and temperature on amylase activities, including thermal and pH stability were discussed. Results showed that out of the 57 isolates with amylase-producing activities, fungi *Aspergillus flavus* 9261 was found to produce amylase with the best activity of 10.7482 U/mg (wet mycelia). The amylase of *Aspergillus flavus* 9261 exhibited remarkable thermostability and pH stability with no activity loss after incubation at 50 °C and pH 5.0 for 1 h, respectively. The results provide advances in discovering enzymes from marine-derived fungi and their biotechnology relevance.

## 1. Introduction

The world’s oceans cover more than 70% of Earth’s area, and are considered to be a great reservoir of diverse microbial communities [1]. Marine microbial communities include bacteria, fungi, viruses, algae, plankton and etc. are essential ecological components in marine environments [2]. They usually play an important role in the biogeochemical process, for example, as intermediaries of energy, as decomposers of dead and decaying organic matter in nutrient regeneration cycles [3]. Thus, marine microorganisms have been attracting more and more attention as a resource for novel molecules and enzymes [4], due to their unique metabolic capabilities. Among marine microorganisms, fungi are a large group of eukaryotic and non-vascular organisms, and are widely distributed in marine environments [5]. Indeed, a great diversity of marine fungi have been recovered not only from seawater and sediment, invertebrates, decaying wood and mangrove detritus, but also as symbionts in marine lichens, plants, and algae [6]. 

Based on the different sources, those microorganisms exclusively from a marine or estuarine habitat are classified as obligate marine fungi, while those from freshwater or terrestrial origin that are able to grow (and possibly sporulate) in marine environments are classified as facultative marine fungi [7]. Nevertheless, the term “marine-derived fungi” is commonly used as a more general classification, due to the fact that most of the fungi isolated from marine samples are not clearly distinguished as obligate or facultative marine microorganisms [3,8]. Studies have demonstrated that marine-derived fungi are ecologically relevant as decomposers of organic matter [9]. In this sense, marine-derived fungi can be considered as a source of hydrolytic and oxidative enzymes with novel physiological characteristics (such as high salt tolerance, thermostability, barophilicity), which can be used in industrial and/or environmental processes. 

Microbial enzymes received great attention from industrial production, and amylase catalyzing the hydrolysis of starch-rich materials is one of the most prominent enzymes [10]. Currently, the amylases mainly used in industrial production (such as sugar, baking, brewing and preparation of digestive aids) are those derived from *Bacillus* strains [11,12]. The optimum activity of those *Bacillus* amylases was reported at a remarkably high temperature and pH optima ranging from 55 to 70 °C, and 3.0 to 11.0, respectively. However, their activity decreases significantly at room or lower temperatures, which limits the development of amylases related industries. Amylases from halophilic producing microorganisms (such as marine microorganisms) may harbor more stability regarding their notable structural features and distinctive functional characteristics [13,14,15]. Among different microorganisms’ sources, marine-derived fungi are the major branch contributing to expanding the number of amylases, especially those with optimum activity at low temperatures [16]. Indeed, versatile amylases have been reported from marine fungi. For example, strain *Aureobasidium pullulans* isolated from the Pacific Ocean sediments was capable of producing exocellular amylases [17]; A novel amylase was isolated from marine *Mucor* sp. [18]; The production of the enzyme α-amylase was first reported in marine *Pseudoalteromonas undina* NKMB 0074 with a wide range of pH (7–11) [19]. Those microbial sources fulfilled the industrial demands and substituted other hydrolysis approaches. Nevertheless, amylases-producing microorganisms are still the major and desirable source for the discovery of novel enzymes [20,21].

Continuing our longstanding interest in the mining of marine microbial enzymes [22,23,24], and in conjunction with our recent interest in the discovery of novel enzymes [25,26], we engaged in the exploration of highly efficient amylase-producing fungal strains from marine habitats. In the present study, 199 fungi were isolated from the sediments collected from the South China Sea, and 57 amylolytic fungi were successfully obtained. Based on the amylase activity measurement, one representative strain was chosen to characterize the amylase properties and stability. It needs to emphasize that in this study, fungal extracellular amylases were assessed qualitatively (by a visual screening for amylolytic fungi in a plate assay). Additionally, intracellular amylase activities were quantified spectrophotometrically within fungal cell lysates (fungal cell-disrupted mycelia).

## 2. Material and Methods

### 2.1. Marine Sediment Samples

**Figure 1:**
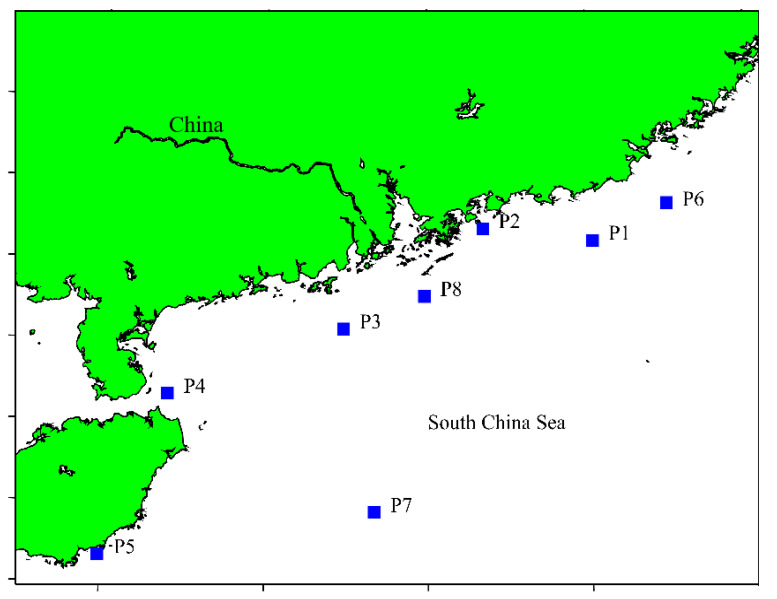
Map of the South China Sea, location of the sampling sites.

Sediment samples at about 200–2492 m depth was collected on the South China Sea Open Cruise in January 2022. Latitudes and longitudes of the collected sediment samples 1–6 are 112.90 °E and 19.48 °N, 112.69 °E and 18.56 °N, 112.63 °E and 18.31 °N, 112.56 °E and 18.00 °N, 112.48 °E and 17.67 °N, 112.36 °E and 17.19 °N, respectively (Figure 1). Sediment sampling was performed using the combination of the Remote Operated Vehicle, a box corer, and an alcohol-sterilized PVC cylinder of 5 cm inner diameter, to keep the collected samples undisturbed and compact. The intubation depth of sediment cores obtained from these locations was about 28–30 cm. After collection, the sediment samples were directedly introduced into sterile plastic bags filled with N_2_ to avoid any aerial contamination [27]. The bags were sealed and then placed in a box filled with ice and quickly transferred to the microbiology laboratory on board and stored at −4 °C for future analyses.

### 2.2. Fungal Isolation

About 1 g of sediment from each location was transferred into a sterile vial for fungi isolation using a flame-sterilized spatula. For the isolation of fungi, a modified particle plating technique reported by Zhang [28] was used. The components of media for fungi isolation contained malt extract agar (MEA), Czapek Dox agar (CDA), glucose peptone starch agar (GPSA), and potato glucose agar (PDA), with a strength of 1/5 to simulate the low nutrient conditions in the seawater [29,30]. To each basic media, 0.5 g/L benzylpenicillin and 0.03 g/L rose bengal were added, avoiding the growth of bacteria. The isolation was performed on the inoculated plates in the dark at a temperature of 30 °C for different times until the fungi could be distinguished. Based on the morphological observations (such as growth characteristics, mycelia, and diffusible pigments), fungal isolates were identified and transferred into new agar plates for pure culture, and for amylase-producing fungal strain screening.

### 2.3. Visual Screening for Amylotyc Fungi

Marine fungi strain isolated above were sub-cultured in Dox-medium for amylolytic fungi isolation on agar plates. The Dox-medium contained (per liter in seawater): NaNO_3_ (2 g), K_2_HPO_4_ (1 g), MgSO_4_·7H_2_O (0.5 g), KCl (0.5 g), FeSO_4_·7H_2_O (traces), and agar (20 g). The final pH was adjusted to 5, supplemented with soluble starch (1% *w*/*v*) and incubated for 72 h at 30 °C. Thereafter, 1% (*w*/*v*) iodine solution was dropped into plates waiting for the amylase to provide clear zones. Experiments were performed in triplicate, and the average values were shown as the final results.

### 2.4. Fungi Identification and Phylogenetic Analysis

The combination of morphological differences and internal transcribed spacersequences (ITS) gave the identification of amylolytic fungi strains. The steps for Fungal ITS gene sequencing and identification are listed below: Total genomic DNA was extracted from all selected fungal strains by using DNA Isolation Kit; From the genomic DNA, nearly full-length ITS sequences were amplified by polymerase chain reaction (PCR) using primers ITS1 (5′-TCCGTAGGTGAACCTGCGG-3′) and ITS4 (5′-TCCTCCGCTTATTGATATGC-3′); The PCR products were sequenced by BGI Technology CO., Ltd. (Shenzhen, China); The sequence results were compared in GenBank by BLAST (Basic Local Alignment Search Tool).

Nearly full-length ITS sequences of amylolytic fungi strains were aligned using Clustal X (1.83) in MEGA 5.0 software, applying the default parameters. Phylogenetic trees of ITS sequences were created using the neighbor-joining (NJ) method with bootstrap analysis using 1000 replicates. This study showed that incorporating fungal sequences with the highest homology of sequences existed in National Center for Biotechnology Information (NCBI).

### 2.5. Amylase Production and Protein Estimation

The obtained fungal spores from a 7-day-old slant culture were sporulated (0.5 cm × 0.5 cm) in flasks with 50 mL aliquots of the sterilized Dox-medium (attuned to pH 5.0). The sporulated mixtures were incubated continuously at 30 °C for 7 days. After filtration through Whatman No. 1 filter paper, the fermented broth was separated, giving the filtrate and the mycelia. The mycelia were collected and washed twice with 100 mM of sodium citrate buffer (pH 5.5) and resuspended in the same buffer. The mycelia were disrupted using a French press (2.05 kBar, 2 shots). Cell-free extract and cell debris were separated by centrifugation for 40 min at 10,000× *g* at 4 °C. The protein content of clear supernatant was determined by Lowry et al. [31], and the dinitrosalyicylic acid (DNS) method [32] was applied for measuring amylase activity. One unit (1 U) of enzyme activity is defined as the number of mycelia (wet weight) catalyzing the hydrolysis of 1 mg starch within 1 min. Experiments were performed in triplicate and the average values were shown as final results. It needs to mention that enzyme activity means the cell-lysate enzyme activities of the cell-disrupted mycelia.

### 2.6. Characterization of Enzyme Stability

The optimum pH was determined by standard activity assay [33] at different pH in the range of 5.0–11.0, with sodium citrate buffer (50 mM) for a pH range from 5.0–6.0, potassium phosphate buffer (50 mM) for a pH range from 6.0–9.0, glycine-NaOH buffer (50 mM) for pH 9.0–11.0. For the determination of the temperature optima, a standard activity assay was performed at different temperatures in the range of 25–75 °C. The reaction mixtures were kept at each temperature for 5 min before starch and crude enzyme solution were added to initiate the reaction. And activity measure at standard conditions (pH 5.5, 25 °C) was taken as 100%. Experiments were performed in triplicate, and the average values were shown as the final results.

In order to determine its thermostability, the crude enzyme solution was incubated at different temperatures (37, 45, 50, 55, 60 and 65 °C) for 1 h, and the residual activity was measured by standard activity assay. The activity of the enzyme without incubation at the given temperature was defined as 100%. In addition, pH stability was recognized according to testing variant gradient buffer values ranging from pH = 2 to pH =13 (for 60 min) at room temperature. Residual activities were examined following the standard conditions. Experiments were performed in triplicate, and the average values were shown as the final results.

## 3. Results and Discussion

### 3.1. Fungal Isolation

We started our investigation with fungi isolation using conventional culture-dependent methods. A total of six marine sediment samples of the South China Sea were processed for fungi isolation on extract agar (MEA), Czapek Dox agar (CDA), glucose peptone starch agar (GPSA), and potato glucose agar (PDA), respectively. Although there is always difficult to recover culturable marine-derived fungi from sediment samples, a total of 199 fungi isolates were successfully obtained in this study (Table 1). The discovery of culturable marine-derived fungi might be due to the use of effective isolation media (MEA, CDA, GPSA and PDA), which have been successfully used for isolating deep-sea fungi from the Central Indian Basin [34] and the South China Sea [28]. To identify the obtained fungi isolates, ITS sequence analysis and observation of morphological characteristics were combined. Although, several isolates had relatively lower similarities to their closest matches, most of the isolates had 95–100% sequence similarities to the existing fungal ITS sequences in the NCBI database. The identified fungi bellowed to 11 different genera, including *Aspergillus*, *Chaetomium*, *Cladosporium*, *Cyphellophora*, *Fusarium*, *Meyerozyma*, *Neodeightonia*, *Penicillium*, *Saccharomyces, Ochroconis* and *Trichobotrys*. Unfortunately, none of the discovered fungi were new from sediment of deep-sea environments, probably due to the fact that a significantly increasing number of fungal communities had been identified with metabarcoding studies. As a result, it is challenging to isolate novel strains from sediment of deep-sea environment based on the method of isolation and cultivation. Nevertheless, it needs to mention that the fungal communities were different from different sampling sites, for example, 41 and 42 isolated were obtained from sites P2 and P4, respectively, while only 10 isolates were obtained from site P7. To achieve a greater diversity of fungal signatures in marine sediments, a combination of culture-dependent and culture-independent approaches might be beneficial, which is under way in our laboratory.

### 3.2. Screening for Amylotyc Fungi (Qualitative Determination of Extracellular Enzyme Activity by Visual Plate Assay)

The ocean is considered to be a great reservoir of biodiversity, leading to bioactive metabolites from marine-derived fungi are considered an attractive point in the field of drug discovery and production. Indeed, there is a number of research highlighting the diversity of fungi derived from different marine environments as well as their potential as producers of bioactive marine natural products. Unfortunately, the use of marine microorganisms as sources of novel enzymes is still rare.

The growing commercial demand for new and more efficient enzymes to implement and optimize industrial processes, which highly depends on new renewable and environmentally sustainable enzyme sources. The adaptability of marine-derived fungi to oceanic conditions makes them an attractive source of unique enzymes. Therefore, we turned our attention to exploring marine-derived fungi from the South China Sea for amylase activity screening. Thus, all the 199 fungi strains isolated above were sub-cultured in Dox-medium for amylolytic fungi isolation on agar plates, as described in Section 2.3.

**Figure 2:**
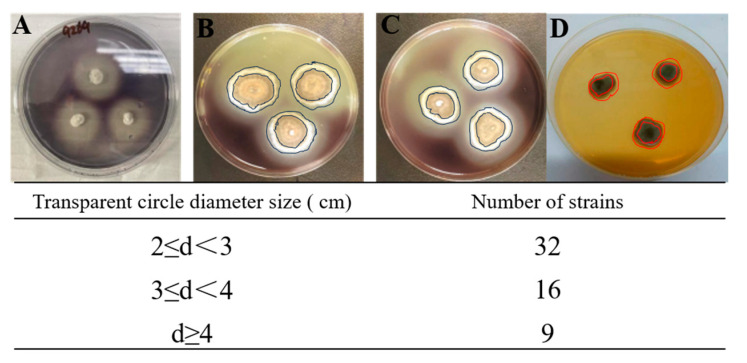
Examples of transparent circles produced by marine fungi with amylase activity, (**A**): fungi with no amylase activity; (**B**,**C**): fungi with amylase activity; (**D**): the use of *Aspergillus fumigatus* as a positive control.

**Figure 3:**
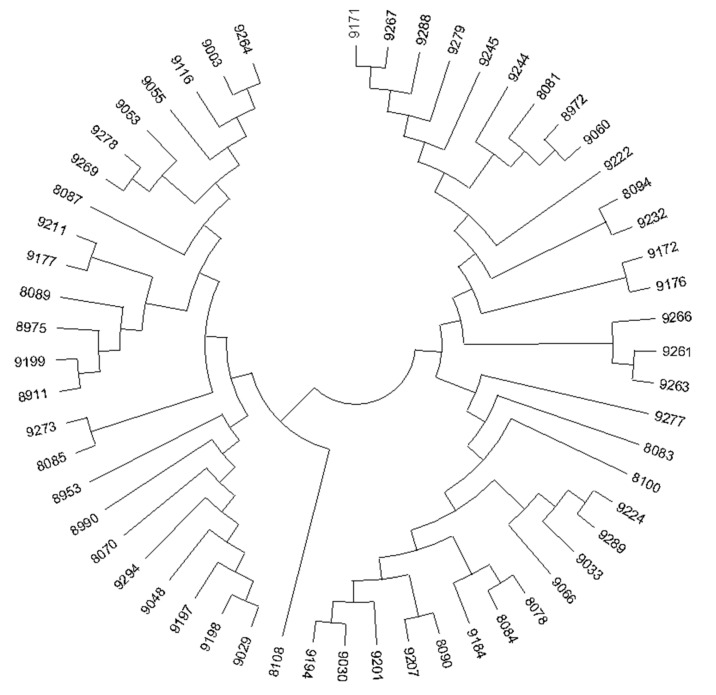
The phylogenetic tree of 57 marine fungi with significant amylase activity. The names of all the fungi are available in Table S1 of the Supporting Information.

The amylase activity was visible as clear zones on the agar plates created by the fungal isolates (Figure 2). The larger the transparent circle produced by marine fungi on the plate containing starch, the stronger the ability of fungi to produce amylase. In this study, only clear zones of a diameter of more than 2 cm were considered as the positive starch zone hydrolysis by tested marine-derived fungi. Gratifyingly, the results of amylase activity screening showed that 57 isolates (Figure 3 and Table S1) showed amylase activity, of which 30 belonged to the genera *Aspergillus*, suggesting that *Aspergillus* sp. are the main sources of amylase-producing fungi. This is supported by the findings of Barakat, who proposed that the genera *Aspergillus* are essential sources of amylases-producing fungi [33]. Indeed, *Aspergillus* species was characterized as the most pervasive and easy to culture microorganisms, Thus, such species are the major and desirable source for the discovery of novel amylases.

### 3.3. Characteristics of Cell-Lysate Enzyme Activity (Quantitative Determination of Intracellular Enzyme Activities Spectrophotometrically)

Encouraged by the discovery of 57 amylase-producing fungi strains, we continuously evaluated the cell-lysate extract enzyme activity toward starch hydrolysis. In order to have a clear idea about the amylase activities of the 57 marine fungi obtained above, the dinitrosalyicylic acid (DNS) method [32] was applied for measuring the amylase activity. The mycelia of the 57 amylase-producing fungi strains were collected and washed twice with 100 mM of sodium citrate buffer (pH 5.5) and resuspended in the same buffer. The mycelia were disrupted using a French press (2.05 kBar, 2 shots). Cell-free extract and cell debris were separated by centrifugation for 40 min at 10,000 rpm at 4 °C. The clear supernatant was collected and used for the measurement of the protein content and the amylase activity. It needs to mention that one unit (1 U) of enzyme activity is defined as the number of mycelia (wet weight) catalyzing the hydrolysis of 1 mg starch within 1 min. It needs to mention that enzyme activity means the cell-lysate enzyme activity of the cell-disrupted mycelia.

The results are summarized in Table 2. Notably, the amylase enzyme produced by marine fungi showed good to excellent activity. The strain *Fusarium nirenbergiae* 9245 produced amylases with a poor activity of 0.062 U/mg towards starch hydrolysis, while the amylase produced by the strain *Aspergillus flavus* 9261 showed the best catalytic activity of 10.7482 U/mg. The observation highlights that *Aspergillus* sp. has been considered the main source of amylases-producing fungi. Notably, *Pezizomycotina* sp. 8081 is a potential source for amylase production of significant starch hydrolysis activity, which was found for the first time. Taking a closer look at the starch-degrading activity of *Aspergillus flavus* 9261, we found that the cell-lysate enzyme activity of the marine *Aspergillus terreus* SS isolated from the Red Sea was shown to be 5.326 U of per mg wet mycelia (intracellular enzyme activities) [35]. As a result, we can make the conclusion that the marine fungi *Aspergillus flavus* 9261 exhibited an activity of 10.7482 U of per mg mycelia, which is superior to *Aspergillus terreus* SS for the production of amylase enzymes. Taking the activity, cultural conditions and available genome sequence into account, we decided to continue to use the marine fungi *Aspergillus flavus* 9261 for all further studies.

### 3.4. Effect of pH and Temperature on Amylase Activities of Aspergillus flavus 9261

**Figure 4:**
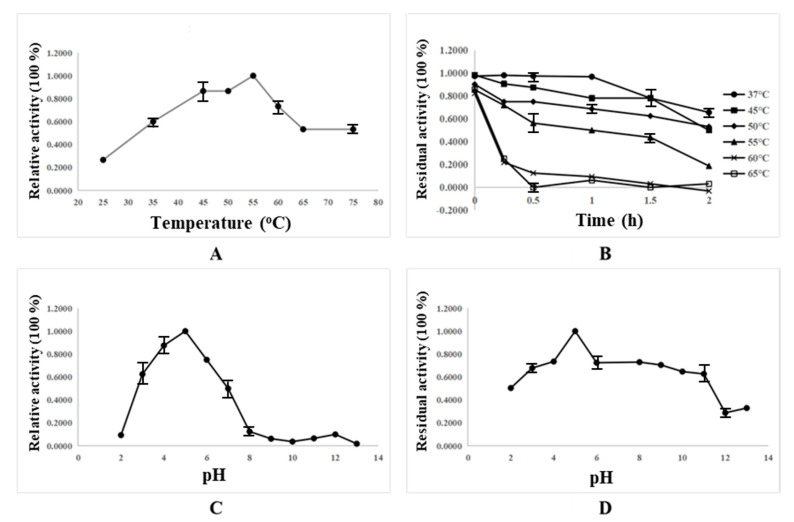
Temperature optima (**A**), thermostability (**B**), pH optima (**C**) and pH stability (**D**) of the amylases produced by *Aspergillus flavus* 9261. (**A**): standard activity assay was performed at different temperatures in the range of 25–75 °C. The reaction mixtures were kept at each temperature for 5 min before starch and crude enzyme solution were added to initiate the reaction. And activity measure at standard condition (pH 5.5, 25 °C) was taken as 100%. (**B**): the crude enzyme solution was incubated at different temperatures (37, 45, 50, 55, 60 and 65 °C) for given times, and the residual activity was measured by standard activity assay. The activity of the enzyme without incubation at the given temperature was defined as 100%. (**C**): standard activity assay was performed at different pH values of 2–11. The reaction mixtures were kept at each pH for 5 min before starch and crude enzyme solution were added to initiate the reaction. And activity measure at standard condition (pH 5.5, 25 °C) was taken as 100%. (**D**): pH stability was recognized according to testing variant gradient buffer values ranging from pH = 2 to pH =13 (for 30 min) at room temperature. Residual activities were examined following the standard conditions. Experiments were performed in triplicate, and the average values were shown as final results.

To fully assess the potential of the marine fungi *Aspergillus flavus* 9261 as amylase-producing strain, the temperature and pH optima was determined. The influence of temperature and pH on the cell-lysate enzyme activity were investigated by monitoring the change in cell-lysate enzyme activity toward starch hydrolysis (Figure 4). For the determination of the temperature optima, a standard activity assay was performed at different temperatures in the range of 25–75 °C. The reaction mixtures were kept at each temperature for 5 min before starch and crude enzyme solution were added to initiate the reaction. Activity measure at standard conditions (pH 5.5, 25 °C) was taken as 100%. Testing a broad range of temperature scales (25–65 °C) revealed that the enzyme exhibited an optimal activity at 55 °C, while it steeply decreased over 60 °C (Figure 4A). On the other hand, testing a broad range of pH scales (2–13) revealed the optimum pH of 5 for the amylolytic activity (Figure 4C). Thus, the amylase produced by *Aspergillus flavus* 9261 was found to have the best activity at pH = 5 and 55 °C, which was supported by the findings (*Aspergillus flavus* AUMC10636 has the optimum activity toward starch hydrolysis at pH = 5.0 and 60 °C) of Ali and workers [36].

In order to determine its thermostability, the crude enzyme solution was incubated at different temperatures (37, 45, 50, 55, 60 and 65 °C) for given times before the substrate was added to initiate the reaction, and the residual activity was measured by standard activity assay. The activity of the enzyme without incubation at the given temperature was defined as 100%. The results showed that the amylase produced by *Aspergillus flavus* 9261 was relatively stable under 50 °C for 1 h, but lost its activity almost completely after incubation at 60 °C for 30 min. In addition, pH stability was recognized according to testing variant gradient buffer values ranging from pH = 2 to pH =13 (for 30 min) at room temperature. Residual activities were examined following the standard conditions. It can be clearly seen that the amylase produced by *Aspergillus flavus* 9261 maintained most of its activity after incubation at pH of 5.0 for 1 h (Figure 4D). Therefore, the ideal condition and incubation period were 50 °C, pH = 5.0 and 1 h, respectively.

## 4. Conclusions

Microbial enzymes received great attention from industrial production, and amylase catalysing the hydrolysis of starch-rich materials is one of the most prominent enzymes. Currently, the amylases mainly used in industrial production are those derived from Bacillus strains. The optimum activity of those Bacillus amylases was reported at a remarkably high temperature ranging from 55 to 70 °C. However, their activity decreases significantly at room or lower temperatures, which limits the development of amylases related industries. Thus, amylases-producing microorganisms (especially fungi) are still the major and desirable source for the discovery of novel enzymes.

The adaptability of marine-derived fungi to oceanic conditions makes them an attractive source of unique enzymes. In this manuscript, we focus on the screening of marine-derived fungi for amylase activity.

In conclusion, this research successfully obtained 199 marine-derived fungi from the South China Sea, of which 57 isolates were found to have amylase activity toward starch hydrolysis by providing clear zones on agar plates. The application of the dinitrosalyicylic acid (DNS) method for measuring the amylase activity revealed that all the tested 57 isolates exhibited an amylase activity of 0.0262–10.7482 U per mg of mycelia (wet weight), which is good to excellent compared to those of reported fungi. The marine fungi *Aspergillus flavus* 9261 was found to produce amylase with the best activity of 10.7482 U/mg (wet mycelia), and is moderately tolerant towards temperature as well as thermal and pH stability. The amylase of *Aspergillus flavus* 9261 exhibited remarkable thermostability and pH stability with no activity loss after incubation at 50 °C and pH 5.0 for 1 h, respectively. In a sense, the potential of amylases produced from marine fungi was considered highly appreciated and of economic value. This opens up an entirely new approach to the discovery of marine-derived enzymes and investigations into their possible applications.

## Figures and Tables

**Table 1:** Diversity and distribution of fungi isolated from six marine sediment samples of the South China Sea.

Fungal Species	Fungal Families	Number of Fungal Isolated							
		P1	P2	P3	P4	P5	P6	P7	P8
*Aspergillus niger*	*Trichocomaceae*		2		2	3	1		1
*A. sydowii*	*Trichocomaceae*	1	2	1	1		2		
*A. terreus*	*Trichocomaceae*		3	2	4	1	1		
*A. oryzae*	*Trichocomaceae*	1	1		2		1	1	
*A. tubingensis*	*Trichocomaceae*		2	1	2			1	1
*A. nomiae*	*Trichocomaceae*	2	1	2		2	1	1	2
*A. flavus*	*Trichocomaceae*		2	1	3	1	2		
*A. versicolor*	*Trichocomaceae*	1			2	1	1		1
*A. phoenicis*	*Trichocomaceae*		1		1				
*A. westerdijkiae*	*Trichocomaceae*		2		2				1
*Chaetomium globosum*	*Chaetomiaceae*		1		1	1			
*Cladosporium cladosporioides*	*Davidiellaceae*	1	1						
*C. colombiae*	*Davidiellaceae*		2		3	3	2		2
*C. oxysporum*	*Davidiellaceae*		2	1	2	1		1	1
*C. sphaerospermun*	*Davidiellaceae*	1	1		3				
*C. uredinicola*	*Davidiellaceae*			3					2
*Cyphellophora fusarioides*	*Cyphellophoraceae*		1		2	1			
*Fusarium proliferatum*	*Nectriaceae*	2		3					
*F. oxysporum*	*Nectriaceae*	1	2	3					1
*F. nirenbergiae*	*Nectriaceae*		2		2	3			
*F. solani*	*Nectriaceae*	2	1		1		1	1	
*Meyerozyma caribbica*	*Debaryomycetaceae*		2	1	1				1
*Neodeightonia subglobosa*	*Botryosphaeriaceae*	1	2		3	2			
*Penicillium aurantiogriseum*	*Trichocomaceae*		1	2	2			1	1
*P. chrysogenum*	*Trichocomaceae*	2	3	3	1	1	1	1	
*P. album*	*Trichocomaceae*		2	1		4		3	2
*Saccharomyces cerevisiae*	*Saccharomycetaceae*	1	1	2	1		2		1
*Ochroconis mirabilis*	*Sympoventuriaceae*		1		1	1	1		
*Trichobotrys effusa*	*Pleosporales*			2		4			1
Total number of fungal isolates		16	41	27	42	29	16	10	18

**Table 2:** The amylase activity produced by cell-lysate extracts from marine fungi ^a,b^.

Fungi	Activity (U/mg)	Fungi	Activity (U/mg)	Fungi	Activity (U/mg)
8070	0.0921 ± 0.0001	9030	0.2790 ± 0.0002	9211	0.1140 ± 0.0001
8078	0.1571 ± 0.0001	9033	0.0964 ± 0.0003	9222	0.0811 ± 0.0002
8081	1.3895 ± 0.0002	9048	0.0880 ± 0.0001	9224	0.0533 ± 0.0004
8083	0.1997 ± 0.0001	9053	0.1793 ± 0.0002	9232	0.3304 ± 0.0001
8084	0.1777 ± 0.0001	9055	0.0499 ± 0.0001	9244	0.1198 ± 0.0001
8085	0.1640 ± 0.0003	9060	0.1140 ± 0.0003	9245	0.0262 ± 0.0003
8087	0.1592 ± 0.0002	9066	0.1146 ± 0.0002	9261	10.7482 ± 0.0002
8089	0.0447 ± 0.0001	9116	0.0293 ± 0.0001	9263	0.5694 ± 0.0001
8090	0.3540 ± 0.0001	9171	0.6806 ± 0.0003	9264	0.2164 ± 0.0001
8094	0.2288 ± 0.0002	9172	0.1606 ± 0.0004	9266	0.3230 ± 0.0001
8100	0.1365 ± 0.0001	9176	0.0723 ± 0.0002	9267	0.4128 ± 0.0002
8108	0.0625 ± 0.0003	9177	0.2946 ± 0.0005	9269	0.1125 ± 0.0001
8911	0.0315 ± 0.00002	9184	0.3517 ± 0.0002	9273	0.0996 ± 0.0002
8953	0.0874 ± 0.0002	9194	0.1654 ± 0.0002	9277	0.1752 ± 0.0001
8972	0.1265 ± 0.0001	9197	0.1629 ± 0.0003	9278	0.1430 ± 0.0001
8975	0.1165 ± 0.0001	9198	0.2528 ± 0.0002	9279	0.2446 ± 0.0002
8990	0.0423 ± 0.0002	9199	0.0641 ± 0.0001	9288	0.2030 ± 0.0003
9003	0.0604 ± 0.0001	9201	0.1991 ± 0.0001	9289	0.4982 ± 0.0002
9029	0.1571 ± 0.0002	9207	0.0668 ± 0.0002	9294	0.1679 ± 0.0002

^a^: One unit (1 U) of enzyme activity is defined as the number of mycelia (wet-weight) catalyzing the hydrolysis of 1 mg starch within 1 min. ^b^: Mean values ± standard deviation of cell-lysate enzyme activity. Experiments were performed in triplicate.

## Data Availability

No data beyond the data presented in the manuscript.

## Supplementary Materials

The following supporting information can be downloaded at: https://www.mdpi.com/article/10.3390/jof9070736/s1, Table S1: Information of the amylase-producing fungus strains.
