# Supplementary material for: Marine-Derived Fungi as a Valuable Resource for Amylases Activity Screening
Source: J Fungi (Basel). 2023 Jul 9;9(7):736. doi: 10.3390/jof9070736 (PMC10381586; doi:10.3390/jof9070736)
Supplement: Supplementary file 1 [file jof-09-00736-s001.zip › jof-2466283-supplementary.pdf]

Supporting Information

Marine-Derived Fungi as a Valuable Resource for Amylases Activity Screening

Di Zhang [1], Lan Liu [1,2] and Bi-Shuang Chen [1,2,*]

[1] School of Marine Sciences, Sun Yat-sen University, Zhuhai 519080, China
[2] Southern Marine Science and Engineering Guangdong Laboratory (Zhuhai), Zhuhai 519080, China
* Correspondence: chenbsh23@mail.sysu.edu.cn; Tel.: +86-0756-3668775

Table S1. Information of the amylase-producing fungus strains

NO.	Fungi	NO.	Fungi
8070	*Aspergillus niger*	9176	*Aspergillus terreus*
8078	*Aspergillus sydowii*	9177	*Aspergillus oryzae*
8081	Pezizomycotina sp.	9184	*Aspergillus niger*
8082	Ascomycota sp.	9194	*Aspergillus* sp.
8084	*Aspergillus* sp.	9197	*Aspergillus niger*
8085	*Aspergillus sydowii*	9198	*Aspergillus* sp.
8087	Pezizomycotina sp.	9199	*Aspergillus nomiae*
8089	*Penicillium chrysogenum*	9201	*Aspergillus flavus*
8090	*Aspergillus terreus*	9207	*Aspergillus terreus*
8094	*Aspergillus niger*	9211	*Aspergillus flavus*
8100	*Aspergillus sydowii*	9222	*Fusarium proliferatum*
8108	*Penicillium aurantiogriseum*	9224	*Fusarium oxysporum*
8911	*Aspergillus* sp.	9232	*Aspergillus* sp.
8953	*Aspergillus niger*	9244	*Parengyodontium album*
8972	*Neodeightonia subglobosa*	9245	*Fusarium nirenbergiae*
8975	*Aspergillus terreus*	9261	*Aspergillus flavus*
8990	*Aspergillus niger*	9263	*Aspergillus flavus*
9003	*Parengyodontium album*	9264	*Parengyodontium album*
9029	*Aspergillus niger*	9266	*Aspergillus* sp.

9030	*Aspergillus oryzae*	9267	*Fusarium oxysporum*
9033	*Fusarium solani*	9269	*Fusarium oxysporum*
9048	*Aspergillus tubingensis*	9273	*Aspergillus versicolor*
9053	*Meyerozyma caribbica*	9277	*Trichoderma* sp.
9055	*Fusarium solani*	9278	*Fusarium oxysporum*
9060	*Neodeightonia subglobosa*	9279	*Fusarium oxysporum*
9066	*Saccharomyces cerevisiae*	9288	*Fusarium oxysporum*
9116	*Parengyodontium album*	9289	*Fusarium oxysporum*
9171	*Fusarium nirenbergiae*	9294	*Aspergillus phoenicis*
9172	*Aspergillus nomiae*		
